# Cost analysis of allogeneic hematopoietic stem cell transplantation before and after the implementation of therapeutic drug monitoring

**DOI:** 10.1016/j.htct.2026.106516

**Published:** 2026-09-28

**Authors:** Joice Zuckermann, Ana Paula Beck da Silva Etges, Bruno Mateus de Castro, Renata Garcia Ruschel, Luciana Rodrigues de Lara, Lucas Bastos Beltrami, Elenita Teresinha Chagas, Liane Esteves Daudt, Alessandra Aparecida Paz, Leila Beltrami Moreira

**Affiliations:** a Hospital de Clínicas de Porto Alegre (HCPA), Porto, Alegre-RS, Brazil; b Universidade Federal do Rio Grande do Sul (UFRGS), Porto, Alegre-RS, Brazil

**Keywords:** Hematopoietic stem cell transplantation, Allogeneic, Time-driven activity-based costing, Therapeutic drug monitoring

## Abstract

**Objectives:**

To estimate the costs of allogeneic hematopoietic stem cell transplantation before and after the implementation of therapeutic drug monitoring.

**Methods:**

A retrospective cohort study with historical controls included adult and pediatric patients undergoing related, unrelated and haploidentical allogeneic hematopoietic stem cell transplantation from December 2014 to December 2021. The therapeutic drug monitoring (cyclosporine, tacrolimus, and busulfan) group (TDM Group) was compared to a Control Group without therapeutic drug monitoring. A microcosting study based on Time-Driven Activity-based Costing was conducted to assess the hospitalization cost of hematopoietic stem cell transplantation patients. The values of medications and materials were adjusted for inflation to December 2022 and expressed in U.S. dollars

**Results:**

Forty-four patients were included in both groups, with data collection from December 2014 to December 2017 (Control Group) and from January 2019 to December 2021 (TDM Group). The average cost per transplantation patient in the entire cohort was US$ 29.604,23 (SD: ± US$ 27.070,00). The hospitalization cost was US$ 32,639.81 (SD ± 31,903.45) for patients in the Control Group and US$ 26,568.65 (SD: ± US$ 21,013.31) for the TDM Group. Regarding the tests performed, the Control Group resulted in a mean cost of US$ 2338.59, with a reduced cost in the TMD Group to US$ 1391.94 (p-value <0.001). The component with the most significant impact on the total cost was medications, with an overall average of US$ 17,728.43 (SD: ± US$ 24,035.77).

**Conclusion:**

The total hospitalization cost for allogeneic hematopoietic stem cell transplantation did not differ significantly between groups, but the TDM Group showed lower exam and medication expenditures, suggesting economic benefits. Despite similar overall costs, therapeutic drug monitoring may provide advantages in other clinical outcomes not assessed in this study. Further research is warranted to explore these endpoints.

## Introduction

hematopoietic stem cell transplantation (HSCT) can be curative for different hematologic diseases, but it involves significant rates of complications, such as graft-versus-host disease (GVHD), infection, and disease relapse, that lead to increased hospitalization costs and even transplant failure. Severe sepsis, acute respiratory failure, endotracheal intubation, and total parenteral nutrition can double the total cost [1]. According to data from the Center for International Blood & Marrow Transplant Research (CIBMTR) [2], >8000 allogeneic HSCTs were performed in the United States in 2020. The median total cost of care estimated for the initial 100 days after allogeneic HSCT in an American cohort study [3] was US$ 289,283 with myeloablative conditioning as opposed to US$ 253,467 with non-myeloablative conditioning and US$ 140,792 for autologous HSCT; the hospitalization period is the most expensive.

Therapeutic drug monitoring (TDM) optimizes the personalized dosing of specific immunosuppressive and chemotherapeutic agents used during conditioning regimens, demonstrating particular relevance in HSCT. The main objective of TDM is to ensure that the plasma concentration of the drug remains within an adequate range, thereby reducing toxicity while maintaining efficacy with better clinical results. Regarding the economic dimension of TDM, it is known that costs associated with clinical outcomes can be avoided [4]. However, there is still little evidence on the benefit of this procedure coordinated by clinical pharmacists in HSCT [5,6]. Although the cost of HSCT has been evaluated across previous studies using diverse costing methodologies and sample sizes, there is a consensus that medications represent the primary component of total expenditures [1,7,8]. In Brazil, there are approximately 140 public and private transplant centers that perform HSCT. These centers are distributed throughout the national territory but are predominantly concentrated in the Southeast and South regions. According to data from the National Transplant System, Brazil performed >22,000 tissue and organ transplants in 2022 alone. Furthermore, the Brazilian Society of Bone Marrow Transplantation and Cellular Therapy reported that 7982 HSCT procedures were recorded between 2012 and 2021 across 31 participating HSCT centers [9]. Microcosting studies to estimate the cost of allogeneic HSCT are scarce in Brazil. The study by Vargas et al. [8] estimated a total cost of US$ 155,110 (Interquartile range: US$ 92,794–249,146) for HSCT.

Microcosting studies assess individual health care costs, allowing us to identify whether cost differences in therapeutic options result from cost variations or the intensity of resource use [10]. Thus, the objective of this study was to estimate the costs of allogeneic HSCT in a patient comparing before and after the implementation of TDM, utilizing the time-driven activity-based costing (TDABC) method.

## Methods

A retrospective cohort study with historical controls and microcosting based on the TDABC method was conducted at the Bone Marrow Transplant Center of the Hospital de Clínicas de Porto Alegre (HCPA), a public university hospital. The study was approved by the institution's research ethics committee (CAAE 08,762,918.1.0000.5327).

Adult and pediatric patients with malignant or non-malignant hematologic diseases who underwent related, unrelated or haploidentical allogeneic HSCT at the center between December 2014 and December 2021 were eligible for inclusion. Patients who received HSCT in 2018 were excluded to minimize the potential influence of the TDM protocol learning curve during its initial implementation. Two cohorts were defined: the TDM Group, consisting of patients who underwent transplantation between January 2019 and December 2021 and received TDM for cyclosporine, tacrolimus, and busulfan; and the historical Control Group, comprising patients transplanted between December 2014 and December 2017, when TDM was not routinely employed. Patient demographic, clinical, and cost-related data were retrospectively collected from electronic medical records and institutional databases by the research team using a standardized data collection form to ensure consistency and accuracy.

In the TDM Group, serum concentrations of cyclosporine, tacrolimus, and busulfan were measured according to institutional protocols. Monitoring frequency followed clinical need, typically daily or every other day during the conditioning and early post-transplant period, and less frequently thereafter. The clinical pharmacists were responsible for calculating pharmacokinetic and pharmacodynamic parameters (area under the curve or trough levels) and recommending individualized dose adjustments. In the TDM protocol, the pharmacokinetic and pharmacodynamic (PK/PD) calculation was applied from the serum dosages of cyclosporine and tacrolimus. The clinical pharmacist recommended the subsequent dose after evaluating potential drug interactions, renal function, serum magnesium and potassium levels, and the manifestation of adverse effects. For busulfan, adjustments were made according to the calculation of the area under the concentration-time curve from time zero to the last quantifiable concentration time point (AUC_0-t_), age, weight, height, body mass index, creatinine percentage, aspartate transaminase, alkaline phosphatase, total bilirubin and alanine transferase, and conditioning protocol. These recommendations were discussed with the medical team during daily multidisciplinary rounds and integrated into the patient’s treatment plan. In the Control Group, dosage adjustments followed the routine practice of the hematology and HSCT service, without incorporating clinical pharmacokinetics. Cyclosporine and tacrolimus trough levels were measured intermittently based on clinical judgment, but no structured TDM protocol, pharmacokinetic assessment, or pharmacist-led dose optimization was implemented.

The costing process followed the TDABC methodology as recommended by the Brazilian Ministry of Health guidelines for microcosting studies. Cost data were obtained from the institution's management system (AGHUSE) with support from the internal financial team. The main cost drivers included personnel time, use of materials, medications, exams, structural resources (partitioned into intensive care unit [ICU] and general ward stays), medical staff and care team, hemodialysis sessions and hemotherapy. The values ​​considered for exams, materials, and medications represent the acquisition cost for the institution without any profit. For hemotherapy, the value of the blood bag was calculated using the values ​​estimated by Magro et al. [11]. The cost structure incorporated all resources utilized during the hospitalization period for HSCT, based on a detailed process map of patient care activities. Time estimates for each personnel time allocation involved in the HSCT care process were obtained through structured interviews with staff members and validated by department supervisors. Time equations were developed to represent the duration and frequency of each activity per patient. To calculate the total time allocated to each activity, the average duration of the intervention was multiplied by the number of times the professional delivered care to each patient. Unit cost rates (capacity cost rates) were calculated for each resource using departmental cost data, productive capacity, and time allocation. The total cost per patient was obtained by multiplying the time consumed by the respective unit cost rate summed across all activities. To estimate the incremental cost of TDM, the pharmacist's activities were divided into: reconciliation, validation, pharmacological review, incompatibilities, conditioning protocol (chemotherapy), TDM and discharge. Following the TDABC method, the following steps were performed: calculation of the cost of each department; definition of standard capacity; calculation of the unit cost rate; identification of the activities developed by each department; processing time estimation for each service; structuring of time equations; and calculation of the cost of services. Institutional financial data from December 2022 were used to determine infrastructure costs and the hourly labor rates of clinical professionals. The values of medications and materials were adjusted for inflation to December 2022 using the Brazilian Broad Consumer Price Index (IPCA) and expressed in U.S. dollars at an exchange rate of R$5.19 per USD (Central Bank of Brazil, December 1, 2022). The analysis was conducted from the hospital’s perspective, considering direct costs incurred by the institution.

## Statistical analysis

Cost data were summarized using means, standard deviations (SD), and medians. Comparisons between groups were performed using non-parametric tests (the Mann-Whitney U test for two-group comparisons and the Kruskal-Wallis test for multiple categories) between the groups and by sex, age and clinical variables within each group. Statistical significance was defined as a p-value <0.05. An analysis of the cost of the input components was performed to assess the impact of the TDM on medications, materials, and tests costs. Missing or incomplete data were checked against original medical records, and no imputation was performed. Outliers were retained in the analysis given their clinical plausibility and relevance to cost variability.

## Results

A total of 88 patients were included in the study, 44 in the Control Group and 44 in the TDM Group, with data collected on hospitalization for allogeneic HSCT in a protected environment unit in the periods from December 2014 to December 2017 and January 2019 to December 2021, respectively. The sample characteristics are described in Table 1. Approximately half of the patients in the Control Group were aged between 10 and 25 years old, while in the TDM Group, this age group represented 25% of the sample, with patients aged 25 years or older predominating. In the Control Group, there were more females than males, while in the TDM group, men predominated. Haploidentical HSCT was performed almost exclusively in the TDM Group. Bone marrow was the most prevalent source of cells in both groups. The primary diagnoses were leukemia in the Control Group and myelodysplastic syndrome and myeloid leukemia in the TDM Group.

**Table 1:** Baseline group characteristics - n (%).

Characteristic	TDM Group (n = 44)	Control Group (n = 44)	p-value
**Gender** (Male)	28 (63.6)	21 (47.7)	0.13
**Age**			0.03
< 10	14 (31.8)	9 (20.5)	
10 to < 25	11 (25.0)	23 (52.3)	
≥25	19 (43.2)	12 (27.3)	
**Type of HSCT**			< 0.001
Haploidentical	17 (38.6)	2 (4.5)	
Matched related donor	11 (25.0)	22 (50)	
Matched unrelated donor	16 (36.4)	20 (45.5)	
**Source**			< 0.001
Bone marrow	28 (63.6)	43 (97.7)	
Peripheral blood stem cells	16 (36.4)	0 (0.0)	
Cord blood	0 (0.0)	1 (2.3)	
**HLA compatibility**			0.50
Partial compatibility	26 (72.2)	22 (64.7)	
Total compatibility	10 (27.8)	12 (35.3)	
No data available	8 (18.8)	10 (22.7)	
**ABO incompatibility**			0.19
Major	9 (22)	13 (40.6)	
Minor	11 (26.8)	5 (15.6)	
No incompatibility	21 (51.2)	14 (43.8)	
**Diagnosis**			0.19
Lymphoid leukemia	14 (31.8)	11 (25)	
Myeloid leukemia	9 (20.5)	10 (22.7)	
Myelodysplastic syndrome	6 (13.6)	7 (7.9)	
Aplastic anemia	1 (2.3)	7 (15.9)	
Others	14 (31.8)	9 (20.5)	
**Length of hospital stay (days)**	54 (21)	60 (41)	0.44

The p-value was calculated using the chi-square test

The total hospitalization cost per patient in the cohort was US$ 29,604.23 (SD: ± US$ 27,070.00); for patients in the Control Group it was US$ 32,639.81 (SD: ± US$ 31,903.45) and US$ 26,568.65 (SD: ± US$ 21,013.31) for the TDM Group. The cost components were grouped as infrastructure, medical supplies, personnel time allocation, hemodialysis, and chemotherapy in Figure 1. The component with the most significant impact on the total cost was medical supplies, responsible for US$ 20,062.39 (69.9%). Among the medical supplies, the most significant portion (88.37%) was due to drugs in both groups, with an overall mean of US$ 17,728.43 (SD: ± US$ 24,035.77).

**Figure 1:**
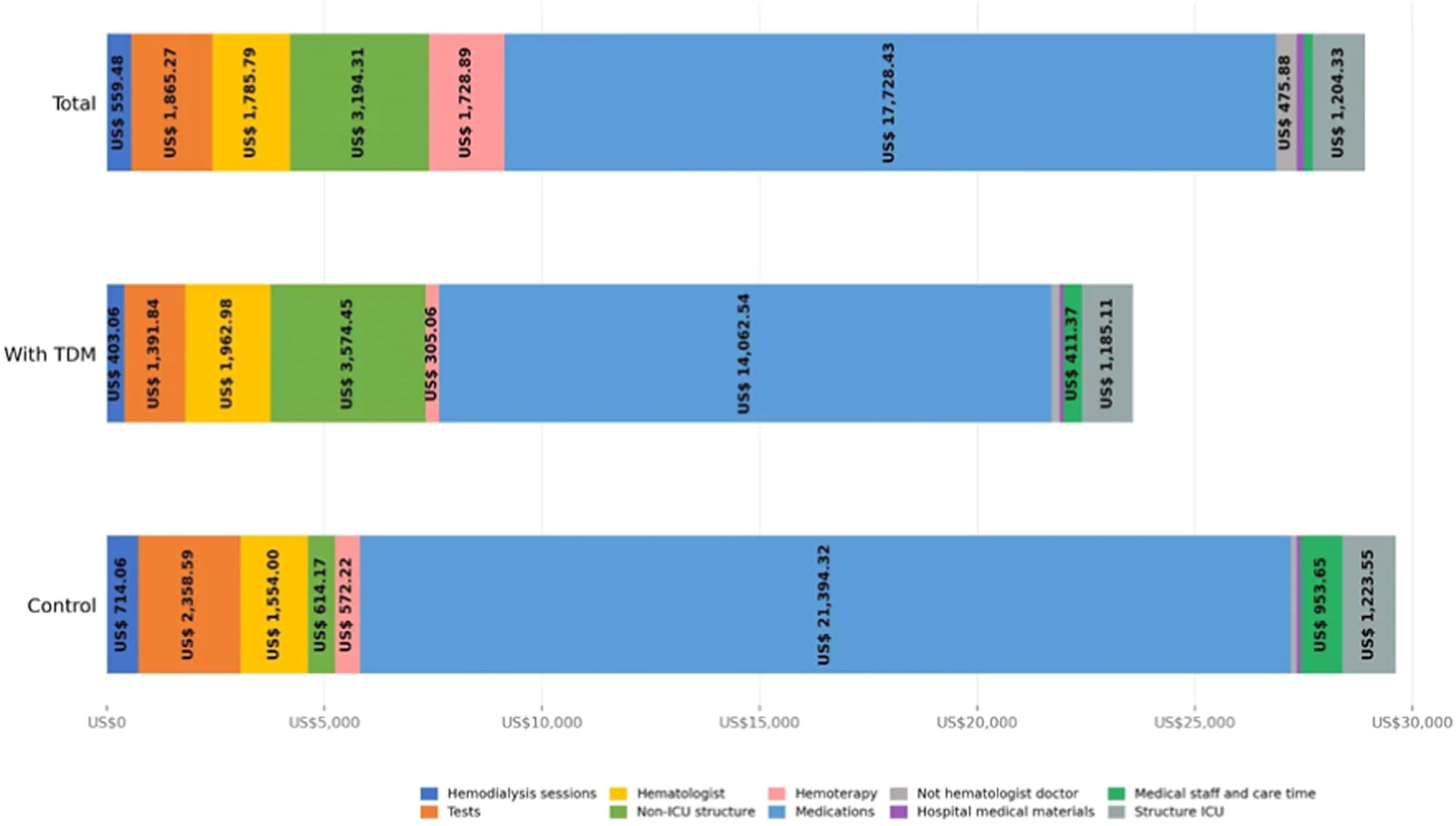
Composition of hematopoietic stem cell transplantation cost before (control) and after the implementation of therapeutic drug monitoring (TDM) of cyclosporine, tacrolimus, and busulfan. (Control Group 2014–2017; TDM Group 2019–2021).

Table 2 shows the cost stratified by demographic and clinical characteristics. The cost by age group did not follow a linear pattern. Unrelated HSCT had the highest cost in the Control Group (p = 0.01), while haploidentical HSCT had the highest cost in the TDM Group (p = 0.66). The total cost of patients who presented acute GVHD did not differ significantly from those who did not have acute GVHD in either group. The average cost of the patient who died during hospitalization was higher than that of those who survived in the TDM Group.

**Table 2:** Average cost (US$) of hematopoietic stem cell transplantation according to demographic and clinical characteristics by study group.

	TDM Group (n = 44)	Control Group (n = 44)
**Total cost per patient**^a^Mean (SD) Median (P25 / P75)	26,568.65 (SD ± 21,013.31) 19,491.78 (12,997.04 / 31,284.32)	32,639.81 (SD ± 31,903.45) 19,588.85 (11,568.83 / 41,757.43)
**Gender**		
Male - Mean (SD)	25,547.70 (± 41,713.79)	34,011.89 (± 28,152.69)
Female	28,355.26 (± 18,765.21)	31,387.00 (± 35,572.98)
p-value	0.34	0.72
**Inpatient age (year)** - Mean (SD)		
< 10	20,590.70 (± 16,644.93)	18,821.03 (± 12,377.07)
10 to < 25	32,518.65 (± 19,765.34)	41,226.36 (± 38,638.41)
≥ 25	27,528.68 (± 24,234.29)	26,546.35 (± 23,035.21)
p-value	0.11	0.12
**Length of hospital stay (days)** - Mean (SD)		
until 38	17,468.96 (± 9312.29)	21,523.85 (± 13,881.80)
39 to 47	44,536.49 (± 35,755.72)	37,273.52 (± 30,428.81)
48 to 65	25,911.27 (± 11,581.99)	38,754.31 (± 48,125.38)
66 to 228	22,385.30 (± 14,439.42)	30,309.59 (± 27,113.36)
p-value	0.14	0.74
**Type of HSCT**^b^ - Mean (SD)		
Haploidentical	30,768.32 (± 22,622.08)	27,147.991 (± 23,639.41)
Matched related donor	26,216.31 (± 28,878.26)	21,252.50 (± 16,852.28)
Matched unrelated donor	22,348.69 (± 11,175.06) d	45,715.04 (± 40,335.16) d
p-value	0.66	0.01
**Source**^c^ - Mean (SD)		
Bone marrow	25,997.99 (± 19,555.15)	32,864.48 (± 32,245.78)
Peripheral blood stem cells	27,317.28 (± 24,515.30)	0
Cord blood	0	22,980.09 (n = 1) d
p-value	0.65	0.91
**Diagnosis** - Mean (SD)		
Lymphoid leukemia	24,435.83 (± 12,872.43)	44,922.86 (± 30,073.25)
Myeloid leukemia	42,745.49 (± 30,356.80)	26,661.70 (± 24,996.90)
Myelodysplastic syndrome	31,071.24 (± 27,471.22)	26,714.79 (± 18,700.08)
Aplastic anemia	53,116.67 (n = 1) d	22,450.79 (± 8966.47)
Others	14,476.07 (± 5510.55)	40,530.78 (± 52,927.16)
p-value	<0.01	0.39
**Death during hospitalization** - Mean (SD)		
Yes	39,801.10 (± 21,099.62)	41,557.63 (± 21,350.97)
No	23,628.08 (± 2011.30)	30,016.92 (± 34,216.22)
p-value	<0.01	0.03
**Graft-versus-host disease (GVHD)** - Mean (SD)		
Acute GVHD		
Yes	26,398.37 (± 20,520.94)	38,051.93 (± 39,901.34)
No	26,792.67 (± 22,209.54)	28,526.60 (± 24,263.40)
p-value	0.36	0.44

a Mann-Whitney U test; p = 0.71.

b Kruskal-Wallis p = 0.027.

c Generalized linear models

d SD not estimated due to number of patients = 1.

Regarding the tests performed (Figure 1), the Control Group resulted in a mean cost of US$ 2338.59, with a lower cost in the TDM Group at US$ 1391.94 (p < 0.001). Cyclosporine and tacrolimus assays were performed in both periods, with a cyclosporine assay cost of US$ 3.50 and a tacrolimus assay cost of US$ 4.30. With the implementation of TDM, the number of assays requested was reduced, which impacted the total cost of exams. Busulfan monitoring based on the area under the curve was introduced in 2018 with a cost of US$ 161.70 per exam. The pharmacist/patient activity value was US$ 37.47 in the Control Group and US$ 126.23 in the TDM Group since new TDM-related activities were incorporated, increasing the pharmacist's time per patient from 234 min to 746 min.

## Discussion

This study analyzes the costs associated with allogeneic HSCT patients treated at a reference university hospital and is, to our knowledge, the first to evaluate the financial impact of TDM managed by a clinical pharmacist in this population. The main finding of this study is that the implementation of TDM in allogeneic HSCT did not significantly change the total hospitalization cost but was associated with meaningful cost avoidance, particularly through reductions in laboratory testing and medication use. In addition, it is the most extensive microcosting study of HSCT conducted with primary data in Brazil, and by the level of data granularity achieved, it describes the composition of costs during the hospitalization period in two groups, identifying the medications as the most expressive cost component in both groups.

Mechanistically, TDM contributes to cost reduction through several pathways: (i) optimization of immunosuppressant and chemotherapeutic dosing, which reduces unnecessary dose adjustments; (ii) individualized pharmacokinetic and pharmacodynamic assessments that decrease the need for repeated drug-level assays; and (iii) mitigation of drug-related toxicities, ultimately reducing downstream expenditures associated with managing complications. The TDM for several drugs, such as immunosuppressants (cyclosporine, tacrolimus) in HSCT, is the only strategy to individualize dosage adjustment since patients' clinical conditions have great variability. The adjustments are made in real-time, taking into account this variability in addition to the serum levels of the drug [12], making it unnecessary to perform some tests that result in personnel time allocation and, consequently, costs.

Although the pharmacist’s workload increased due to the incorporation of TDM-related activities, this did not translate into higher overall costs, as the savings in exams and medication consumption offset the additional cost of personnel time. TDM-activities contributed, for instance, to avoiding around 40% of exams compared to the Control Group. In addition, individualized TDM enhances patient safety and reduces clinical complications [5], thereby preventing the incremental expenditures required to treat these adverse events.

The findings of this study align with previous literature demonstrating the economic value of individualized pharmaceutical care in HSCT. Vargas et al. [8] conducted a microcosting study with 24 patients in one private and one public hospital in the south of Brazil. The estimated cost of the complete care cycle in allogeneic HSCT was US$ 155,110 (US$ 92,794–US$ 249,146). Their study included both pre-transplant and post-transplant phases and mixed public and private institutions, which likely explains their higher values compared to the present analysis that used a larger sample and was focused exclusively on the inpatient phase in a public hospital setting. International data from Shah et al. [13] also indicate that inpatient care is the major cost driver of HSCT, accounting for 80%–90% of total expenditure, which ranged from €230,399 to €290,125 per patient. These data are consistent with the current findings indicating that medication and infrastructure resources dominate overall costs. The study by Beyron et al. [6] showed that the average total costs per patient were significantly lower in a pharmaceutical intervention group (€85,947 ± €27,003) versus a non-intervention group (€100, 435.6 ± €28,831; p < 0.01), supporting the premise that optimized drug management can generate measurable financial benefits. No economic evaluation studies of allogeneic HSCT were found that analyzed the costs of HSCT according to patients' demographic and clinical characteristics.

The difference in total cost did not reach statistical significance in the present study, possibly due to sample size and the impact of variability on the study power. In the analysis by type of HSCT, there was a cost reduction for unrelated HSCT in the TDM Group compared to the Control Group. This difference may be attributed to the lower number of drug dosages associated with TDM. Baseline differences observed between groups, particularly regarding donor type and stem-cell source, reflect the evolution of clinical practice at the study center over time. During the period corresponding to the Control Group, haploidentical transplantation and the use of peripheral blood as a stem-cell source were still uncommon due to limited institutional experience. These differences reflect temporal changes in practice rather than selection bias but should still be acknowledged as potential confounders in cost comparisons.

This study was not designed to quantify or compare clinical outcomes between groups, but rather to estimate hospitalization costs for allogeneic HSCT using a TDABC microcosting approach. However, some clinical variables indirectly associated with resource use (and therefore cost) appear in the dataset. The length of stay category showed the expected pattern that longer stays tended to be associated with higher costs in both groups (Table 2). Median length of stay did not differ significantly between groups (54 vs. 60 days; p = 0.44), which helps to explain why total costs were not substantially different despite the cost avoidance related to tests and medications observed in the TDM Group. Costs for patients with and without acute GVHD did not differ significantly in either of the study groups. While this variable was included as descriptive context, the study was not powered to detect cost differences stratified by complications.

Mortality during hospitalization was low in both cohorts, and descriptive data indicate that patients who died had higher average costs, particularly in the TDM Group (US$ 39,801 vs. US$ 23,628). This is consistent with the greater intensity of care at the end of life, considering the ICU setting. As the mortality rate was similar across periods, mortality is unlikely to have introduced bias into the cost comparison between the groups. Mortality during hospitalization was included in the total cost because costs were calculated for the entire HSCT admission episode regardless of outcome. There was no censoring or imputation, as TDABC microcosting captures all resources consumed during hospital stays. Mortality was treated as a descriptive variable and not used as an adjustment factor in statistical tests given the study design.

This study has limitations. Although it is the largest microcosting study in Brazil, the sample size is modest, which may have reduced the statistical power to detect differences in total costs. Additionally, the analysis did not include detailed clinical outcome data, limiting the ability to correlate cost changes with potential improvements in toxicity, engraftment, or survival. Although we used standardized cost-year adjustments, inflationary effects and price variability across institutions may influence generalizability. Retrospective data collection for the Control Group may also have led to conservative cost estimates. Finally, because the study was conducted in a single public referral hospital, extrapolation to private centers or other health systems should be made with caution.

An additional limitation concerns the characterization of the Control Group. Although the intervention period was defined by the implementation of a structured pharmacist-led TDM program with pharmacokinetic-pharmacodynamic assessments and individualized dose recommendations, cyclosporine and tacrolimus assays were employed in both study periods. Therefore, differences between groups should be interpreted as reflecting the implementation of a comprehensive TDM service rather than the mere availability of drug concentration measurements. This should be considered when interpreting the magnitude of the observed economic impact.

## Conclusion

There is no significant difference in the total cost of hospitalization for allogeneic HSCT with and without TDM by the pharmacist, but it is possible to identify cost avoidance related to exams and medications in patients under TDM care. By reporting cost information at a micro-level, this study can inform the decision-making processes of healthcare systems and organizations regarding the adoption of these services while considering their implications on clinical outcomes.

## Conflicts of interest

None.

## Data Availability

The data that support the findings of this study are available from the corresponding author upon reasonable request.
